# Chitosan Containing Nano Zn-Organic Framework: Synthesis, Characterization and Biological Activity

**DOI:** 10.3390/polym14071276

**Published:** 2022-03-22

**Authors:** Mohamed Gouda, Hairul-Islam Mohamed Ibrahim, Amr Negm

**Affiliations:** 1 Department of Chemistry, College of Science, King Faisal University, Al-Ahsa 31982, Saudi Arabia; anegm@kfu.edu.sa; 2 Biological Science Department, College of Science, King Faisal University, Al-Ahsa 31982, Saudi Arabia; himohamed@kfu.edu.sa; 3 Biochemistry Division, Chemistry Department, Faculty of Science, Mansoura University, Mansoura 35516, Egypt

**Keywords:** chitosan nanocomposites, zinc–organic framework, biological activity, biopolymer

## Abstract

A biologically active agent based on a Zn-1,3,5-benzen tricarboxylic acid (Zn-BTC) framework incorporated into a chitosan (CS) biopolymer (Zn-BTC@CS) was successfully synthesized using a microwave irradiation technique. The synthesized Zn-BTC@CS was characterized using a scanning electron microscope (SEM) and the obtained data indicated a highly smooth surface morphology of the synthesized Zn-BTC and no morphological changes when the Zn-BTC covered the CS. In addition, the particle size diameter varied from 20 to 40 nm. XRD displayed a well-maintained Zn-BTC structure, and the crystal structure of Zn-BTC was not distorted by the composition of Zn-BTC and chitosan in the nanocomposite. Data from BET analysis revealed that the specific surface area of the Zn-BTC was reduced from 995.15 m^2^/g to 15.16 m^2^/g after coating with chitosan. The pore size distribution and pore volume of the Zn-BTC, Zn-BTC@CS were centered at 37.26 nm and at 22.5 nm, respectively. Zn-BTC@CS exhibited anticancer efficacy against lung and colon cancer cell lines. Zn-BTC@CS inhibited the proliferation of A549 and DLD-1 cancer cell lines in a dose-dependent manner with IC_50_ values of 13.2 and 19.8 µg/mL for the colon and lung cancer cell lines, respectively. Zn-BTC@CS stimulated the apoptotic process through up-regulating P53 expression and down-regulating Bcl-2 expression. Moreover, Zn-BTC@CS induced in vitro DNA fragmentation in both cancer cell lines with significantly different affinity by 66% (A549) and 20% (DLD-1) versus 52% reduction by Cisplatin. Zn-BTC@CS (IC50) exhibited anti-invasive activity and dramatically inhibited the migration of lung and colon cancer cell lines. This study provides evidence that Zn-BTC@CS targets the essential proteins involved in proliferation, metastasis, and apoptosis. Thus, Zn-BTC@CS has chemotherapeutic potential for inhibiting lung and colon cancer viability and growth.

## 1. Introduction

Cancer is a leading cause of death around the world, and the number of new cases and deaths is increasing quickly [1]. Cancer is characterized by uncontrolled cell growth, irregular cell spread, and apoptosis deficiency, all of which lead to death if not regulated [2]. The high burden of cancer has led to the search for safe and effective chemotherapeutic agents. Chemotherapeutic agents are currently the preferred option for cancer treatment through necrosis and/or apoptosis [3]. There is a high demand for novel multitarget anticancer agents that affect various molecular targets to induce cancer cell death.

Porous materials have been the subject of extraordinary interest for quite a while because of their potential applications in drug delivery systems, separating gases, and catalysis [4]. Crystalline materials containing pores are ideal for use in these applications. There are three kinds of permeable materials, and they are characterized by the spans of their pores. These are microporous, mesoporous, and macroporous materials. The pore size influences the material’s properties and its reasonableness for use in various applications.

Metal atoms self-assembling with organic bridging ligands form metal–organic frameworks (MOFs). These ligands are organic compounds such as tricarboxylic acids, pyridyl groups, and imidazoles. MOFs have numerous appealing properties, for example, differing topology, low thickness, high porosity, a tunable pore size of atomic measurements, and high surface areas [5]. They are additionally effectively functionalized through changing the metal or organic compound. The presentation of organic parts in these porous materials likewise enriches the materials with expanded hydrophobicity. These properties make them the subject of high interest for various applications, e.g., gas storing and separation, which are becoming progressively more significant because of ecological concerns [6], and further biomedical applications such as multiple drug resistance [7] and drug delivery vehicles [8]. A broad scope of trial conditions is utilized to combine MOFs and different permeable materials. Solvothermal routes, which use ordinary electric heating, have been generally utilized in the combination of permeable materials [9,10]. Furthermore, to reduce the synthesis time and raise the energy efficacy, new systems using microwave irradiation have been established [11,12,13,14,15,16,17].

Lately, MOFs have obtained broad consideration for use in drug delivery [18,19]. For drug delivery, they have the upsides of high pore surface area, customizable pore measurement, high medication stacking, long-haul supported release, and great biocompatibility [20,21,22,23]. Furthermore, modified MOFs provide a promising nanoscaled drug delivery system for anti-tumor drugs [22]. Associated with additional porous materials, MOFs exhibit numerous exceptional benefits, for instance, (1) high surface area and porosity for high packing of beneficial agents and (2) superficial alteration of the physical (e.g., pore size and shape) and chemical properties of MOFs through inorganic groups and/or organic ligands. Shunzhi et al. reported recent advancements in the synthesis and postsynthetic surface functionalization of MOF NPs that deepen the fundamental knowledge of how such structures form and grow. In addition, the internal structure and external surface properties of these new nanomaterials are also discussed, as are recent advancements that MOF NPs are being used as components in chemical sensors, biological probes, materials for membrane separation, and as building blocks for colloidal crystal engineering as a result of these fundamental developments [24]. Furthermore, a simple templated interfacial synthesis process for the fabrication of a variety of 1-D MOF nano- and microstructures with precisely controlled shapes and sizes has also been developed. Specifically, we construct a zeolitic imidazolate framework-8 (ZIF-8) and ZIF-67 nano- and microstructures with diameters ranging from 10 nm to 20 m using track-etched polycarbonate (PCTE) membranes as templates and at the oil/water interface [25].

A polymer covering over MOFs is an exciting technique to overcome the disadvantages of MOFs, such as their poor biocompatibility, instability, short dissemination time, and rapid degradation. Numerous polymers, such as chitosan [26], could be wrapped around MOFs. Chitosan is a polysaccharide that is generally utilized in biomedical applications [27,28,29,30] due to its biocompatibility and biodegradability [31]. As MOFs are made up of various multivalent metal species and organic ligands, polymers with functional groups, such as –C=O and –COOH, may provide coordination connections with such metal species in MOFs [26]. Its disintegration in acidic pH has attracted considerable attention from scientists for its usage in various biomedical fields [32]. Furthermore, chitosan’s cationic properties make it suitable for drug delivery systems and the regulated release of drugs. Physicochemical properties of chitosan compounds spotlight their functions in passive and active targeted drug delivery, cellular absorption, and tumor growth inhibition (TGI) [33,34]. Besides this, nanoparticles covered with chitosan have great colloidal strength and biodegradability [26].

Using green nanoparticle synthesis, a κ-Carrageenan/Mg0.99Zn0.01O/NaCMC hydrogel was created. Separately, the nanoparticle and polymer were produced and studied. After that, catechin-loaded polymers were made and their physicochemical properties were investigated [33]. The undoped and Zn-doped MgO nanostructures were created using a sol–gel process in a gel environment to get the morphology for the undoped and Zn-doped MgO nanostructures, resulting in a new nanocomposite with acceptable characteristics for biomedical applications [34].

The aim of this study was to use a microwave technique to synthesize mesoporous nanomaterials of a Zn-BTC framework incorporated into a chitosan biopolymer (Zn-BTC@CS). SEM, XRD, and FTIR were used to characterize the synthesized Zn-BTC@CS. Furthermore, the antiproliferative activity, cytotoxicity, and anticancer efficacy of the synthesized Zn-BTC@CS nanoparticles were evaluated against lung and colon cancer cell lines. DNA fragmentation and markers of key genes involved in apoptosis (p53 and Bcl-2) were measured to investigate the mechanism(s) of action by which the nanoparticles exerted their effect.

## 2. Materials and Methods

### 2.1. Materials

Chitosan (85% deacetylated) with an average molecular weight of 100,000 g/mole was obtained from (Merck-Sigma, Darmstadt, Germany). Zinc nitrate hexahydrate (Zn(NO_3_)_2_·6H_2_O) 99.8%, 1,3,5-benzene tricarboxylic acid 98.99%, absolute ethanol 99%, and 98% acetic acid were purchased from (Merck-Sigma, Darmstadt, Germany). 

### 2.2. Synthesis of Zn@BTC/CS Nanocomposite

A volume of 2 mL of 1% chitosan was prepared by dissolving 1 g chitosan in 1% acidic acid and impregnated into a conical flask containing 90.42 mg Zn(NO_3_)_2_·6H_2_O in 12 mL EtOH and 202.0 mg BTC in 12 mL EtOH. The mixture was kept with shaking overnight at 25 °C for 24 h using a Bench-top Shaker and then placed in a microwave oven (power, 1800 W; frequency, 2455 MHz) and exposed to microwave irradiation at 100 °C for 5 min (MARS 6, CEM, Matthews, NC, USA). The irradiated samples were washed several times with ethanol and dried in an oven at 70 °C for 1 h.

### 2.3. Characterization

The surface morphologies of pure CS, Zn-BTC, and Zn-BTC@CS nanocomposite were examined via SEM-JOELF (Tokyo, Japan). In a scanning electron microscope (SEM), a low-energy electron beam is radiated to the sample and scans the sample’s surface. As the beam approaches and enters the material, several interactions occur, resulting in the emission of photons and electrons from or near the sample surface. The samples are adhered with 40–60 nm by gold coater, and then examined under a microscope. An accelerating voltage of 15.0 kV for the SEM image analysis was used. FTIR spectra were recorded using an FTIR-8400S Spectrometer (SHIMADZU, Kyoto, Japan) in the range of 400 to 4000 cm^−1^. The crystal structures of the CS, Zn-BTC, and Zn-BTC@CS nanocomposite were measured by XRD (Rigaku, Tokyo, Japan) using Cu Kα radiation (λ = 0.154 nm) at 40 kV and 30 mA with a 2θ range from 5° to 80°. The d values of the mean diameter of the samples were calculated according to the Scherrer equation [35]: d = k_λ_/(βcosθ), where d is crystallite size, k is a constant applied as 0.89, λ is the X-ray wavelength, β is the full width at half-maximum, and θ is the Bragg angle. 

### 2.4. Specific Surface Area

The measurement of N_2_ adsorption–desorption was calculated on a programmed gas sorption analyzer (Ji Nan RunZhi Technology, Shanghai, China). The prepared samples were outgassed for 4 to 6 h and N_2_ adsorption–desorption isotherms were evaluated at 77.35 K by a volumetric method which represents extra adsorption isotherms [36]. The specific surface area of the prepared samples was determined from isotherm via the Brunauer–Emmett–Teller (BET) procedure. 

### 2.5. Biological Activity Evaluation

#### 2.5.1. Cell Culture and MTT Assay

The MTT tetrazolium reduction assay [37] was the first cell viability assay designed for high-throughput screening in a 96-well format. A colon cancer cell line (DLD-1) and lung cancer cell line (A549) were grown in DMEM using 96-well plates, according to Mosmann, T. [37]. Cells were incubated for 24 h with serial dilutions of Zn-BTC@CS (10–100 µM/mL). Then, cells were washed. The MTT substrate was applied to cells in culture and incubated for 2 h at 0.5 mg/mL. MTT is converted into a purple formazan substance by viable cells with an active metabolism. A plate-reading spectrophotometer was used to calculate variations in the absorbance at 570 nm; color formation serves as a useful and practical indicator of only viable cells. 

#### 2.5.2. Scratch Wound Healing (Migration) Assay

In a 6-well plate, cells were grown in a complete medium and allowed to adhere overnight. After the cells had reached confluence, a line was drawn down the center of the wells with a sterile tip; the wells were gently washed with serum-free DMEM and then treated with either DMSO or Zn-BTC@CS-containing DMEM for another 24 h [37].

#### 2.5.3. Invasion (Transwell Assay)

The invasion test was conducted using a matrigel-coated Boyden chamber (8 μm PET, Corning, NY, USA) [38]. In the upper chamber, cells were cultured in serum-free DMEM with Zn-BTC@CS. In the lower chamber, DMEM with 10% FBS was added. After incubation for 36 h at 37 °C, the upper chamber was cleaned using a cotton swab to clean out the cells that were not invaded. The invasive cells were routinely processed by fixing in 4% formalin for 10 min and then stained in 0.1% Geimsa stain for 15 min, and five randomly selected fields were captured via microscopy at 200× magnification.

#### 2.5.4. DNA Fragmentation Assay

This test is used to examine the cleavage of DNA, as mentioned previously [39,40]. Briefly, cells were exposed to different concentrations of Zn-BTC@CS for 1 day. The cells were collected, washed, and lysed in lysis buffer at 55 °C for 2 h. After DNA extraction and precipitation, the DNA pellet was dried and dissolved in TE buffer containing RNaseA, and DNA fragmentation was evaluated on agarose gel. The gel was stained with Ethidium Bromide (Bioneer, Daejeon, South Korea) and documented under a UV transilluminator.

#### 2.5.5. RT-qPCR

RNA was isolated according to Li, H. et al. [41]. cDNA was synthesized from RNA using reverse transcriptase (TaKaRa Biotechnology, Otsu, Japan). P53 and BCL-2 transcript expression levels were determined using the Applied Biosystems Real-Time PCR system. qPCR was performed in a thermocycler (Applied Biosystems, Foster, CA, USA) with a reaction volume of 10 μL containing 0.03 μg complementary DNA product, 2 μM forward and reverse primers, and the SYBR^®^ FAST qPCR reagent (Kapa Biosystems, Wilmington, MA, USA). The primers used were as follows: BCL-2 “F-GCCGGTTCAGGTACTCAGTCATC” and “R-GTCACCTTCACCGTTCCA”; P53 “F-ACCTATGGAAACTACTTCCTGAAA” and “R-CTGGCATTCTGGGAGCTTCA”; and B actin “F-TCTGCTGGAAGGTGGACAGT,” and “R-CCTCTATGCCAACACAGTGC.” The process comprised 40 cycles of 95 °C for 5 s and 60 °C for 34 s. β-actin was included as a reference control. The comparative 2^−ΔΔCt^ method was used to calculate the relative expression of each gene [42].

## 3. Results and Discussion

### 3.1. Scanning Electron Microscopy 

**Figure 1:**
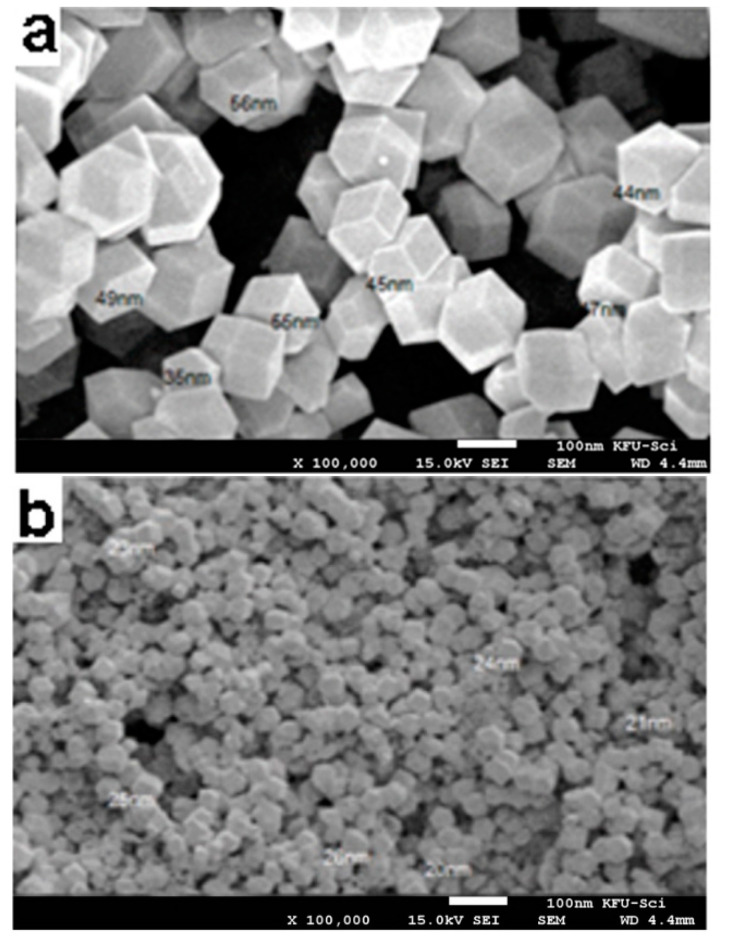
SEM images of (**a**) Zn-BTC and (**b**) Zn-BTC@CS.

The morphologies of Zn-BTC and Zn-BTC@CS nanocomposite were viewed via SEM. Zn-BTC crystals were uniform with an octahedral shape (Figure 1a). Zn-BTC@CS nanocomposite showed a uniform characterized morphology (Figure 1b). Homogeneous circulation of Zn-BTC on CS framed a necklace-like structure. It likewise produced a material with a high surface area and particle diameter around 22 nm.

### 3.2. X-ray Diffraction

**Figure 2:**
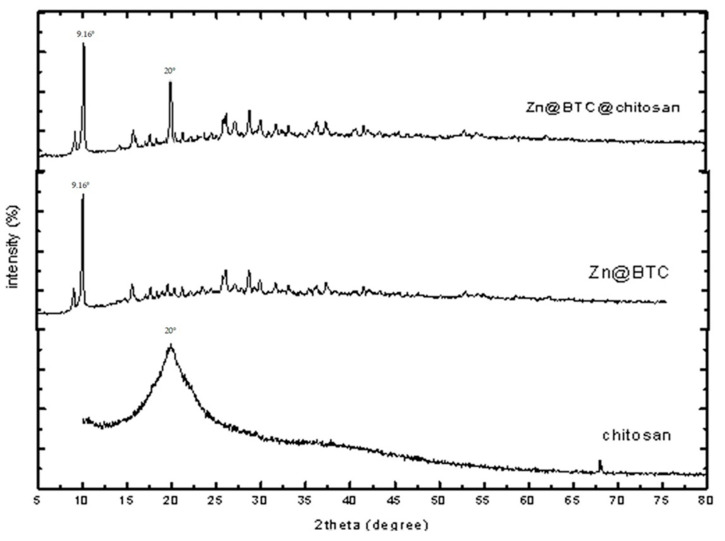
XRD patterns of chitosan, Zn-BTC, and Zn-BTC@CS.

Figure 2 displays the XRD patterns of the CS, Zn-BTC, and Zn-BTC@CS nanocomposite. The significant increase in layer spacing with a high degree of orientation of Zn-BTC was evidenced by a border peak at 2*θ* = 9.16°, which is obvious in the spectrum. The broad reflection at a 2*θ* value of about 20° in the Zn-BTC@CS XRD pattern is related to the amorphous nature of chitosan. The XRD pattern of Zn-BTC@CS is also identical to that of Zn-BTC, indicating that the Zn-BTC structure was preserved. The diffraction patterns are well indexed with the reference code (JCPD card no. 234578). The peaks corresponding to the powder material Zn-BTC and Zn-BTC@CS matched well with the reference pattern, indicating that the Zn-BTC crystal structure was obtained and not distorted. The composition of Zn-BTC and chitosan in the Zn-BTC@CS nanocomposite did not distort the crystalline structure of the Zn-BTC component, according to the XRD results.

Furthermore, the relative strengths and peak places of the example are in concurrence with the review and confirm the development of Zn-BTC. As shown in Figure 2, examination of the XRD peaks of Zn-BTC and the Zn-BTC@CS nanocomposite demonstrates that Zn-BTC powder was present at the chitosan surface. It revealed that Zn-BTC was definitely embedded in the chitosan lattice and that, after washing with ethanol, the nanoparticles did not detach from the chitosan surface.

### 3.3. FTIR

**Figure 3:**
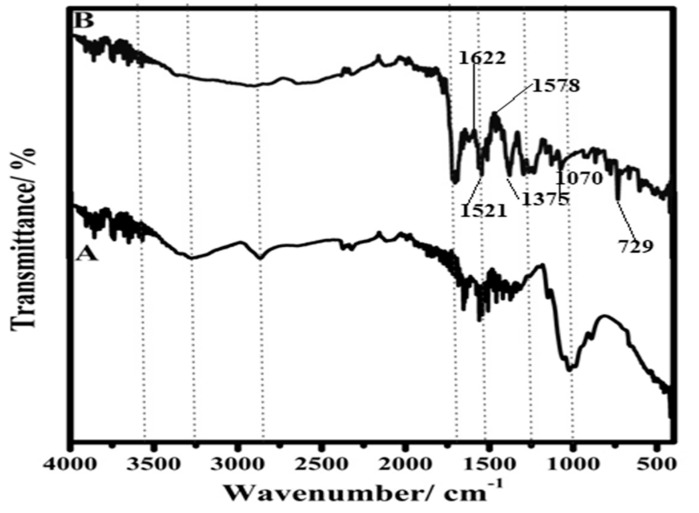
FTIR spectra of (**A**), chitosan and (**B**)Zn-BTC@CS.

The FTIR spectra of blank chitosan and Zn-BTC@CS are shown in Figure 3A,B; the spectra indicate the absorption bands of the carbonyl (C=O) and hydroxyl (–OH) groups. The peak at 1622 cm^−1^ corresponds to the C=O stretching band, whereas the peak at 1375 cm^−1^ corresponds to the stretching vibration of the C–O bond, exhibiting carboxylate deprotonation and coordination with the Zn(II) metal ion to create ZnO_3_(BTC)_2_. The MOF’s distinctive peaks were found at 1578 cm^−1^, which corresponded to the aromatic ring’s C=C, and 1521 cm^−1^, which corresponded to the strong carbonyl group’s C=O. In addition, the presence of the carboxyl group resulted in a distinct peak at 1375 cm^−1^. Due to the shift of the –OH group caused by the presence of carboxylate, no peak was found at 1722 cm^−1^. This is the primary reason for the formation of long conjugate bonds from the carboxylate produced by the carboxyl anion, resulting in the two-oxygen-atom equivalent. Furthermore, a prominent distinctive signal matching ZnO was found at 729 cm^−1^. 

### 3.4. Specific Surface Area

**Figure 4:**
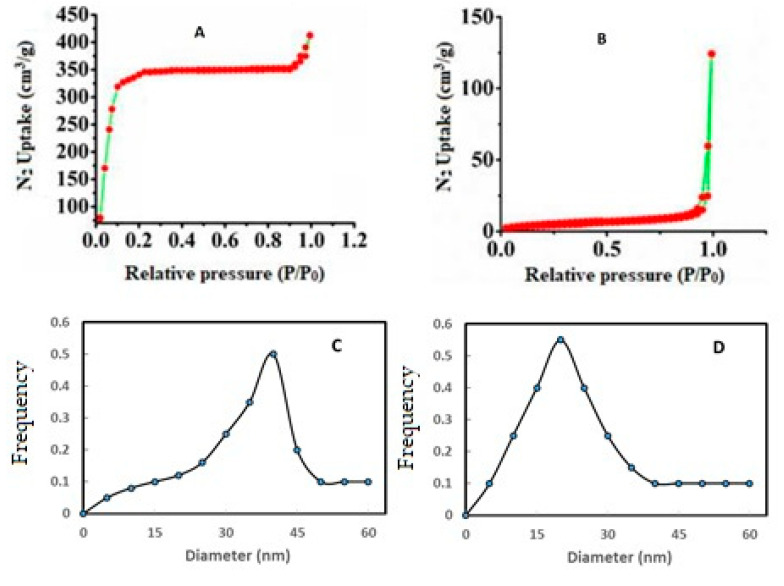
N_2_ adsorption–desorption isotherm at 77.35 K, of (**A**) Zn-BTC, (**B**) Zn-BTC@CS and particle size distribution of (**C**) Zn-BTC, (**D**) Zn-BTC@CS.

N_2_ adsorption–desorption tests were used to investigate the structural properties of prepared samples. The isotherm curve for Zn-BTC is type I, whereas it is type II for Zn-BTC@CS, as illustrated in Figure 4. The type I curve signifies microporous materials, while the type II curve characterizes mesoporous materials, according to the IUPAC organization. Figure 4A shows that, at relative pressures of 0.0–0.90 cm^3^/g, N_2_ adsorption–desorption by Zn-BTC was sluggish, but surged above 0.90 cm^3^/g, reaching a maximum of 410 cm^3^/g. N_2_ adsorption–desorption proceeded slowly in Zn-BTC@CS at relative pressures of 0–0.96 cm^3^/g, then rose to a maximum of 120 cm^3^/g as shown in Figure 4 B. The specific surface area of the Zn-BTC reduced from 995.15 m^2^/g to 15.16 m^2^/g after coating with chitosan, according to the data obtained. Pore diameters and pore volumes of Zn-BTC, (D) Zn-BTC@CS are shown in Figure 4C,D. The pore size distribution and pore volume of the Zn-BTC, Zn-BTC@CS were determined using the Barrett–Joyner–Halenda method. Zn-BTC was centered at 37.26 nm and Zn-BTC@CS at 22.5 nm in the distribution. The values obtained for the surface areas confirm the Zn-BTC covered with chitosan [43] and characterize the particle size distribution of mesopores.

### 3.5. Biological Activity Evaluation

#### 3.5.1. Anticancer Evaluation

**Figure 5:**
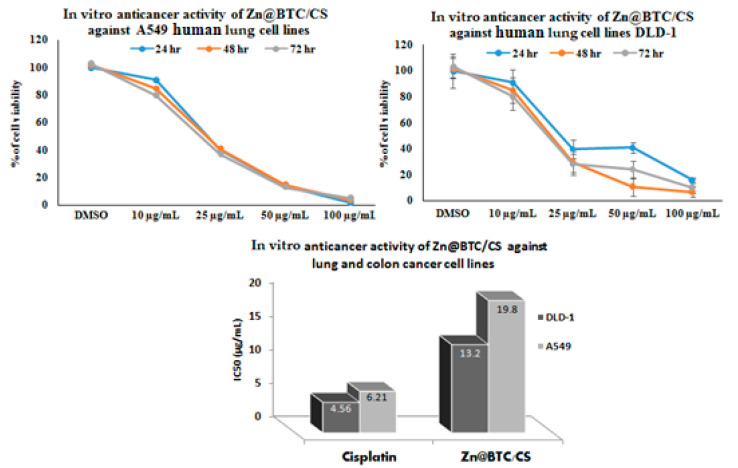
In vitro anticancer activity of Zn-BTC@CS against lung and colon cancer cell lines.

The MTT test is a form of viability test used to screen candidate molecules’ cellular effects to gain a deeper understanding of the cellular processes that can be influenced by known chemotherapeutic agents [44]. The cytotoxicity of Zn-BTC@CS was assessed using the MTT assay. When lung and colon cancer cell lines were exposed to Zn-BTC@CS for 24, 48, and 72 h, the number of viable cancer cells was reduced, with the most notable effect appearing against colon cancer cell lines (Figure 5). Previous studies declared that chitosan-derivative-based nanostructures showed markedly increased therapeutic impact compared to chitosan alone [45]. The MTT test showed the toxic effect of Zn-BTC@CS, as it inhibited the proliferation of A549 and DLD-1 cancer cell lines in a dose-dependent manner with IC_50_ values of 13.2 and 19.8 µg/mL for the colon and lung cancer cell lines, respectively (Figure 5).

#### 3.5.2. DNA Fragmentation Assay

**Figure 6:**
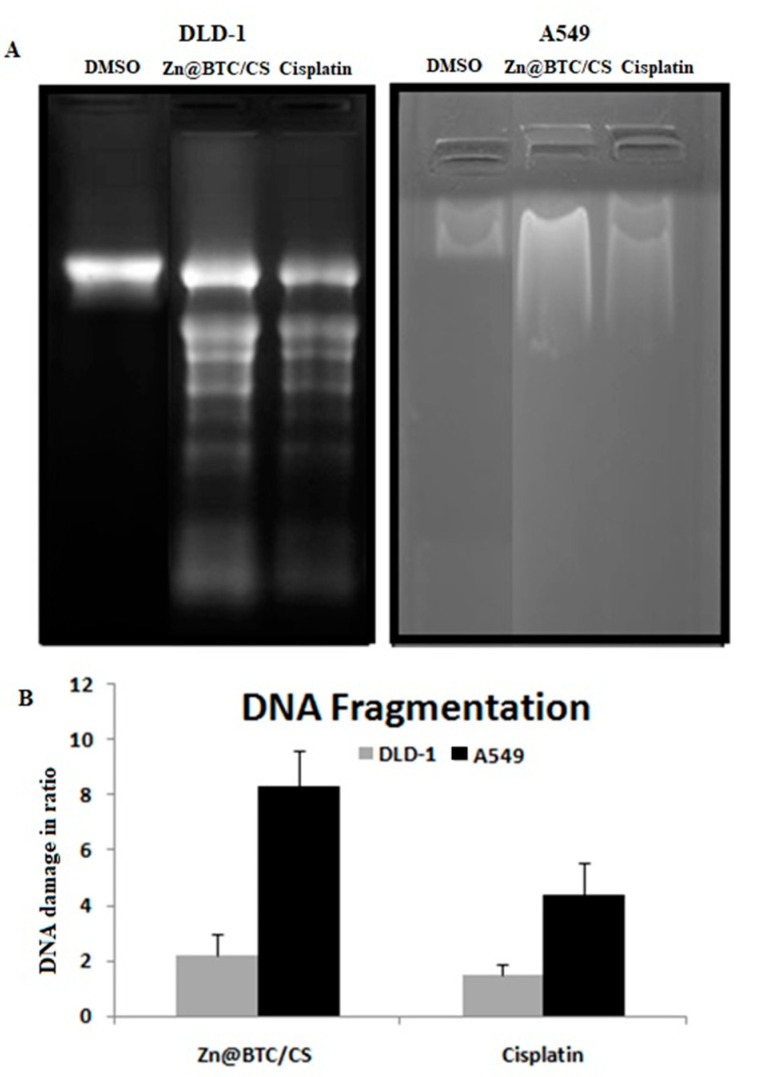
The DNA fragmentation effect of Zn-BTC@CS on lung and colon cancer cell lines showing (**A**) DNA gel electrophoresis and (**B**) DNA damage plot. Values are expressed as the mean  ±  S.D. of 3 independent experiments.

The DNA fragmentation effect of Zn-BTC@CS was evaluated and compared to that of Cisplatin. In Zn-BTC@CS-treated cells, a dose-dependent DNA laddering pattern was identified via nuclear DNA fragmentation assay (Figure 6). Zn-BTC@CS was found to cause a significant elevation in DNA fragmentation in both the lung and colon cancer cell lines by 66% and 20%, respectively, versus a 52% reduction by Cisplatin. The DNA fragmentation assay verified that Zn-BTC@CS is a potent apoptotic agent that triggers nuclear DNA fragmentation into nucleosomal units, which is a hallmark of apoptotic cell death [46].

#### 3.5.3. qRT-PCR Assessment of the Expression of p53 and Bcl-2

**Figure 7:**
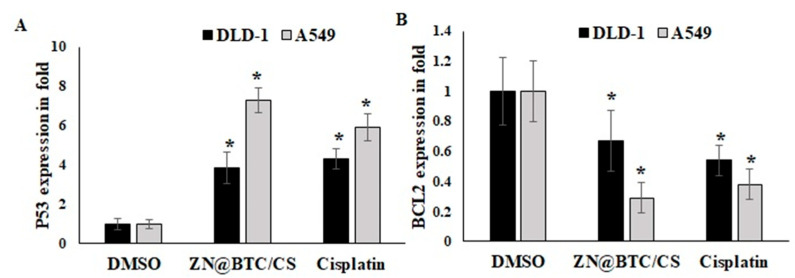
Effect of Zn-BTC@CS on the relative gene expression of (**A**) P53 and (**B**) BCl-2 in lung and colon cancer cell lines. * refers to significant change vs. DMSO treatment.

Apoptosis is a programmed cell death process that keeps cells in a healthy survival/death balance. Apoptosis defects can lead to cancer, while increased apoptosis can lead to degenerative diseases. Apoptotic signals help to preserve genomic integrity. On the other hand, if apoptosis is deficient, this promotes cancer. Thus, modulation of apoptosis is a successful approach in cancer treatment [47]. The effect of Zn-BTC@CS on the expression of key genes involved in apoptosis, such as p53 and Bcl-2, was evaluated. The influence of Zn-BTC@CS on the expression of apoptosis-related genes exhibited up-regulation of p53 and down-regulation of BCL-2 in a dose-dependent manner (Figure 7). The p53 gene is essential for inducing programmed cell death and maintaining genomic integrity [48]. Increased p53 expression improves cancer cell chemotherapeutic sensitivity [49], while BCL-2 antagonizes the apoptotic event [50]. Therefore, several anticancer drugs target BCL-2/BAX-dependent pathways [51]. Zn-BTC@CS was found to cause significant reductions in Bcl-2 expression in both the lung and colon cancer cell lines by 66% and 20%, respectively, versus a 52% reduction.

#### 3.5.4. Migration

**Figure 8:**
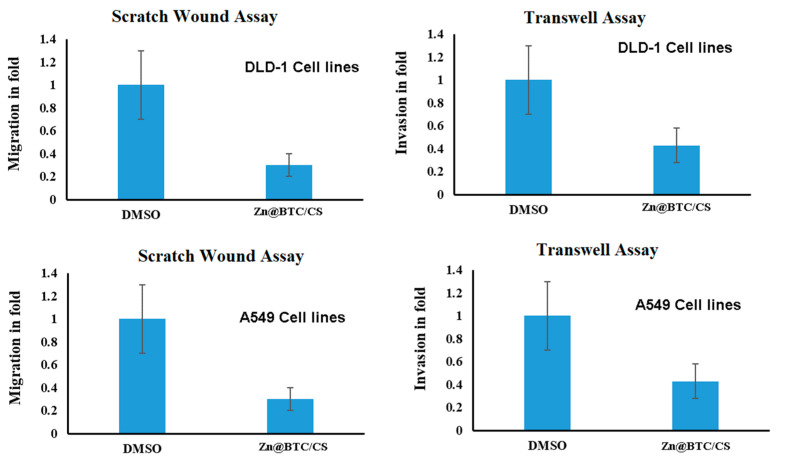
Anti-invasive and -migrative effects of Zn-BTC@CS on lung and colon cancer cell lines.

The wound-healing assay was performed using Zn-BTC@CS-treated lung and colon cancer cell lines to further explore whether Zn-BTC@CS affects human cancer cells’ motility. Anti-healing properties are the key factor aside from apoptosis for controlling cancer progression. In cancer prognosis, migration and adhesion are associated [52]. Treatment of cells with Zn-BTC@CS (IC50) significantly inhibited migration (Figure 8), and it occurred more slowly than it did for cells in the control group (0 μmol) at 36 h. Zn-BTC@CS-treated cells delayed the wound healing time in DLD-A549 cell lines and inhibited cell motility in a manner related to migration marker breakdown. This was validated by further quantitative real-time PCR. Our results indicate that Zn-BTC@CS showed a potential anti-metastatic effect on DLD-1 and A549 D cells.

#### 3.5.5. Invasion

The effect of Zn-BTC@CS on cell invasiveness capability was investigated using the Boyden chamber assay. The number of invaded cells among DLD-1 and A549 cells was reduced by 50%. The binding of fibronectin to integrins in the cell migration process is important for cancer cell invasiveness [53,54]. The inhibition of cellular acceleration gives rise to potential anticancer effects. Furthermore, cancer cells’ proliferation characteristics cause adhesion and migration [55]. As a result, the inhibitory effects of Zn-BTC@CS on BCl-2 in the colon and lung cancer cells and up-regulated expression of P53 show that the treated molecule provides anti-apoptotic and anti-metastatic effects via the contribution of anti-invasive and -migrative effects.

## 4. Conclusions

Mesoporous nanomaterials of a Zn-1,3,5-benzentricarboxylic acid framework incorporated into chitosan biopolymer were successfully synthesized using a microwave technique. SEM images revealed that the surface morphology of synthesized Zn-BTC is highly smooth, and no morphological changes were observed when the CS was covered with the nano-MOFs. In addition, the particle size diameter varied from 22 to 50 nm. The XRD results showed that the Zn-BTC structure was well maintained, and the crystal structure of the Zn-BTC was not distorted by the combination of Zn-BTC and chitosan in the Zn-BTC@CS nanocomposite. Zn-BTC@CS nanocomposite treatment reduced cell viability by inhibiting cell proliferation and development and caused apoptosis and DNA fragmentation in lung and colon cancer cells, suggesting that it could be used as an anticancer agent to inhibit tumor progression.

## Data Availability

The authors confirm that the data of this study are available within the article. Raw data are available from the corresponding author upon request.
